# Supplementary material for: Pathophysiological Significance of Neutrophilic Transfer RNA-Derived Small RNAs in Asymptomatic Moyamoya Disease
Source: Cells. 2021 May 1;10(5):1086. doi: 10.3390/cells10051086 (PMC8147334; doi:10.3390/cells10051086)

2D Structure

```
263                                 292
                            8mer-1a
5'-gctCGAAGCACCCAGGTTGC-CCACGGAAAa-3' utr
      ||  :  |  |||  :|:|  | |||||||
3'-uuuGCACUAUUGGU--GAUGUGAUGCCUUUg-5' tRF
             16  151413     7 6 5 4 3 2
           3' Pairing          Seed
```

Local AU

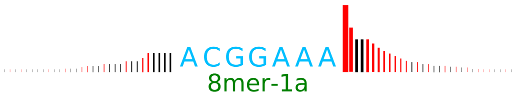

Position

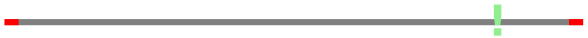

Supplement: Supplementary file 1 [file cells-10-01086-s001.zip › cells-1149161 3rd supplementary materials/Data Supplements/Data Supplement V.zip!/Data supplement 6-mRNAs targets of tsRNAs/tsRNA-Val-AAC-017/Diagrams/tRF-Val-AAC-017_vs_ENST00000357485(ARID5A).pdf]
